# Reducing the diagnostic delay in Antiphospholipid Syndrome over time: a real world observation

**DOI:** 10.1186/s13023-021-01906-1

**Published:** 2021-06-16

**Authors:** Massimo Radin, Silvia Grazietta Foddai, Alice Barinotti, Irene Cecchi, Elena Rubini, Savino Sciascia, Dario Roccatello

**Affiliations:** 1 Center of Research of Immunopathology and Rare Diseases-Coordinating Center of Piemonte and Valle d’Aosta Network for Rare Diseases, and SCDU Nephrology and Dialysis, Department of Clinical and Biological Sciences, S. Giovanni Bosco Hospital, University of Turin, Piazza del Donatore di Sangue 3, 10154 Turin, Italy; 2 School of Specialization of Clinical Pathology, Department of Clinical and Biological Sciences, University of Turin, Turin, Italy; 3 Nephrology and Dialysis, Department of Clinical and Biological Sciences, S. Giovanni Bosco Hospital, University of Turin, Turin, Italy

**Keywords:** Antiphospholipid Syndrome, Diagnostic delay, Rare diseases, Antiphospholipid antibodies

## Abstract

**Background:**

Antiphospholipid Syndrome (APS) is a rare autoimmune disorder with an estimated prevalence of 40–50 cases per 100.000 persons. Patients suffering from low prevalence diseases are more likely to face diagnostic challenges, given the limited knowledge of most clinicians. The main aim of this study was to investigate the time between symptoms occurrence and the diagnosis of APS patients using the Piedmont and Aosta Valley Rare Disease Registry. Secondly, to evaluate the individual impact of the diagnostic gap by gathering patients’ personal experiences through a self-administered questionnaire.

**Results:**

Data from the Piedmont and Aosta Valley Rare Disease Registry was used. In addition, personal experiences were analyzed through a self-administered questionnaire. A total of 740 APS patients included in the Piedmont and Aosta Valley Rare Disease Registry were analyzed. Diagnostic delay (as defined by time between symptoms’ occurrence and the diagnosis of APS) was significantly reduced over time. In particular, when comparing the diagnostic delay between patients diagnosed between 1983 and 1999 and patients diagnosed between 2000 and 2015, we found a significant statistical difference (Mann-Whithey U Test; mean rank 1216.6 vs. 1066.9, respectively; p < 0.0001). When analyzing the self-administered questionnaires, patients with a perception of having suffered for a diagnostic delay had a higher prevalence of symptoms suggestive of an autoimmune condition but not highly suggestive of APS (45%), followed by “extra criteria” APS manifestation (30%) and by thrombotic events (25%). The first clinical manifestation of patients who did not have the perception of having suffered a diagnostic delay was thrombotic events (45.5%), followed by autoimmune manifestation not linked to APS (45.5%), and “extra criteria” APS manifestations (9%).

**Conclusions:**

While the diagnostic delay of APS has been reduced during the last years, the time between symptoms occurrence and the diagnosis of rare diseases still represents a critical issue to be addressed in order to prevent major complications.

## Introduction

Antiphospholipid Syndrome (APS) is an autoimmune disorder characterized by thrombotic events and/or pregnancy morbidity (PM) in the presence of persistent presence of antiphospholipid antibodies (aPL) [1]. While APS is often referred as the most common acquired thrombophilia, the global incidence of APS is estimated to be 5 cases per 100,000 persons per year, with an estimated prevalence of 40–50 cases per 100,000 persons [2–4]. Consequently, APS is listed among the rare diseases, defined according to both the European (prevalence less than or equal to 5 cases per 10,000 people) [5] and US (any disease or condition that affects fewer than 200,000 people in the United States or about 1 in 1500 people) definitions [6].

Recent studies [7, 8] have highlighted the limited knowledge of most clinicians when they face rare pathological conditions. Consequently, patients suffering from low prevalence diseases are more likely to face diagnostic challenges, with the risk of encountering under diagnosis or delayed diagnosis. This might severely impact APS patients, as they might experience recurrence of thrombosis and/or PM episodes while waiting for a diagnosis and consequently an appropriate treatment.

The objective of our study was to analyze the diagnostic delay (as defined by time between symptoms occurrence and the diagnosis of APS) based on data from the Piedmont and Aosta Valley Rare Disease Registry and by gathering patients’ personal experiences through a self-administered questionnaires.

## Methods

### Registry

Data were collected from the Piedmont and Aosta Valley Rare Disease Registry, as part of the National Registry of Rare Diseases, by the coordinating Center of Piedmont and Aosta Valley Network for Rare Diseases, S. Giovanni Bosco Hospital, Turin, Italy. The Registry is based on a continously implemented  dataset, including socio-demographic and disease data, as detailed elsewhere [11].

A total of 740 patients with a definite diagnosis of APS has been included in the Rare Disease Register of Piedmont and Aosta Valley over the last 35 years. Data of the Registry include age at first clinical manifestation of the patient and age of the patient at diagnosis.

## Diagnostic delay questionnaires

A self-administered questionnaire was appositely designed to enquire the diagnostic delay in APS. The questionnaire was administered to APS patients fulfilling the current diagnostic criteria [1] and meeting the following characteristics: (1) with an active follow-up at the San Giovanni Bosco Hospital; (2) APS diagnosis within the last 5 years.

Due to a retrospective character of the study, there was a risk that the information given by the respondents might not be exact. Therefore, for this part of the study, the authors took into consideration only patients who had been diagnosed within the last 5 years preceding the study or were undergoing diagnosis. This allowed to reduce the influence of time on the quality of the respondents information.

Questionnaires were filled out anonymously by the patients via Google Forms. All patients signed an informed consent. This study was conducted in compliance with the Declaration of Helsinki and was approved by the local ethical committee.

## Results

### Registry

Data from a total of 740 APS patients included in the Piedmont and Aosta Valley Rare Disease Registry was analyzed. Median age at diagnosis was 45 years old (Interquartile Range, IQR 34–57), while the median age of the first clinical manifestation of APS (either thrombotic or pregnancy morbidity) fulfilling the diagnostic criteria of the disease [1] was 40 years old (IQR 29–52). The mean diagnostic delay was of 4.7 years (S.D. ±8.3), median 1 year (IQR 0–5).

**Fig. 1:**
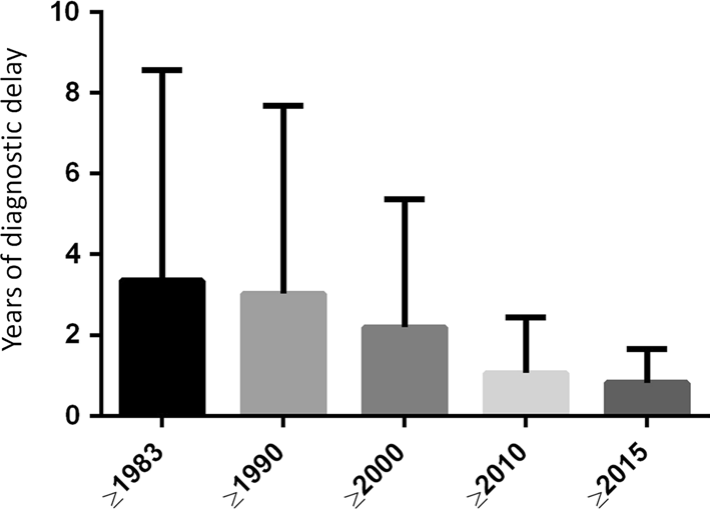
Box plot of the diagnostic delay of antiphospholipid syndrome stratified by time period

Data was then stratified by time period, as shown in Fig. 1. Interestingly, the diagnostic delay was significantly reduced over time, starting from data collected since 1983 (the year of APS description [10]), with a mean diagnostic delay of 3.4 years ± 5.2 (median 5 years; IQR 3–10), since 1990 (mean diagnostic delay 3 years ± 4.7; median 5, IQR 3–9), since 2000 (mean diagnostic delay 2.2 years ± 3.2; median 4, IQR 2–7), since 2010 (mean diagnostic delay 1.1 years ± 1.4; median 3, IQR 3, 2-3.75) and since 2015 (mean diagnostic delay 0.8 years ± 0.8; median 2, IQR 2, 2–3).

When comparing the diagnostic delay between patients diagnosed between 1983 and 1999 and patients diagnosed between 2000 and 2015, we found a significant statistical difference (Mann-Whithey U Test; mean rank 1216.6 vs. 1066.9, respectively; p < 0.0001).

### Questionnaire

Among the eligible patients, thirty-three patients (66% females; mean age at data collection 46.7 ± 13.2 years old) filled out the anonymous questionnaire via Google Forms. The rate of response to the questionnaire was (33/74, 45%). Seventeen patients (52%) were primary APS, while 16 patients (48%) had secondary APS. Twenty-four patients (73%) had at least one thrombotic event, 6 patients experienced pregnancy morbidity (3 recurrent miscarriages and 3 foetal deaths), and 3 patients fulfilled the criteria for both thrombotic and obstetric APS.

When asked if, in their perception, there has been a delay when diagnosing their autoimmune condition, 20 patients (61%) replied yes, 11 (33%) replied no and 2 (6%) were unsure of the reply. Of the patients that thought there had been indeed a diagnostic delay, the mean delay was of 46.5 months (S.D. ±65; max 240; min 3). When we asked these patients if their clinical manifestations, in light of their current disease knowledge, were suggestive of APS, the majority of case (50%) answer yes, while 30% were unsure and 20% believed that their first clinical manifestations were not linked to APS.

Interestingly, when looking at first clinical manifestations of autoimmune disease, the most common manifestation in patients that believed there had been a diagnostic delay was an autoimmune manifestation not linked to APS, as arthralgia and cutaneous manifestations (45%), followed by “extra criteria” APS manifestations, such as thrombocytopenia, hip osteonecrosis, migraine and *livedo reticularis* (30%) and by “criteria” manifestations such as thrombotic events (25%). In comparison, the first clinical manifestation of patients that did not believe there had been a diagnostic delay were “criteria” manifestations, such as thrombotic events and pregnancy complications (45.5%), followed by autoimmune manifestation not linked to APS (45.5%), and “extra criteria” APS manifestation (9%).

When we focused on patients with referred diagnostic delay (20/33) and analyzed only those with a diagnostic lag of more than one year (8/20), we noticed that patients that waited the most (namely patient 1 and 2) were those with symptoms that emerged in the nineties, when APS knowledge was growing and sharpening. More in detail, patient 1 manifested first clinical sign in 1990 and was diagnosed in 2015; patient 2 had first clinical sign in 1998 and was diagnosed in 2016. In case 1, the first symptom was a cutaneous rash, that was later classified as systemic lupus erythematosus over time; in case 2 amnesia, migraine and digital petechiae manifested as first clinical signs, all now recognized “extra criteria” manifestations of APS.

**Fig. 2:**
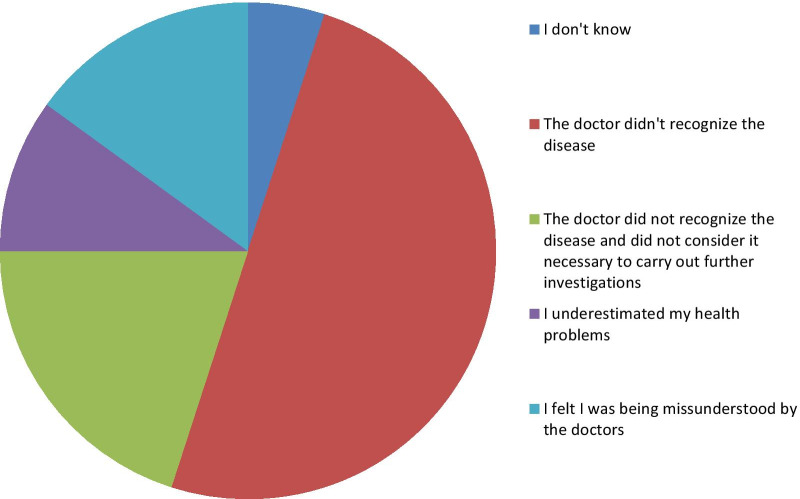
Relative representations of reasons listed by the patients of the delay in diagnosing their condition

When enquiring what was the reason the patients believed the delay was caused by, the majority of patients (16/20, 80%) thought that the doctor that first diagnosed them had been wrong or didn’t recognize the disease proceeding with further investigations. Two patients claimed of being misunderstood by the clinician and of having neglected their health issues contributing to the delay. All the reported answers are listed and resumed in Fig. 2.

When exploring the number of clinicians/centers consulted before diagnosis was established, 26 patients (79%), with and without declared delayed diagnosis, reported of being evaluated by one to three different physicians. Seven patients (21%), nonetheless, consulted more than four different centers/physicians before receiving APS diagnosis. Respondents stated that the first clinician who suspected the disease was a specialized physician, but three subjects indicated their general practitioners as the first clinician to point it out.

## Discussion

The delay of diagnosis represents one of the main challenges in the field of rare diseases. Patients affected by these conditions often have to wait months or years before having the right diagnosis. As previously reported, this is primarily due to a lack of knowledge of most clinicians, that are thus unable to recognize these pathologies, and also to a lack of time to work on these diagnoses [7]. The consequences on patients’ conditions are serious. An early diagnosis of any rheumatic disease indeed has a significant impact on the effectiveness of treatment, the reduction of disease activity and the chance of remission. For instance, in patients affected by rheumatoid arthritis there is a strong evidence that a prompt diagnosis (in particular within three months from the symptoms onset) is associated with an improvement of the clinical and radiographic outcomes and also with a higher rate of remission [11].

Given this, in the last years, several attempts have been made in order to overcome the diagnostic delay in rare diseases, for instance the development of diagnosis support tools, automatic algorithms or methods based numerical scores [7].

With regard to APS, the analysis of the Rare Disease Registry of Piedmont and Aosta Valley highlighted a mean diagnostic delay of 4.7 years, that shortened to less than one year when considering the period 2015–2020.

These results were confirmed by the use of the self-administered questionnaires, which underlined the persistence of a temporal gap between the occurrence of first clinical sign and diagnosis, even if gradually reducing. It has to be noticed that both “criteria” and “extra criteria” manifestations were correlated with diagnostic delay [12]. Indeed, patients with “extra criteria” manifestations and especially those who are positive for *non criteria* aPL, such as anti-phosphatidylserine/prothrombin antibodies, more likely face a delay in diagnosis or are at higher risk of been misdiagnosed [13].

As well as for the other rheumatic diseases, the implications of a prompt APS diagnosis are multiple. An early diagnosis allows rapid treatment and the reduction/avoidance of recurrent thrombotic and PM events, in patients that potentially are very young, impacting both on quality of life and permanent disability. Furthermore, correct identification of rare diseases patients is crucial as these patients might have the necessity of dedicated health plans, specialized care in tertiary care centers or dedicated exemptions from contribution to the healthcare expense.

Given this, even if it has been observed a reduction in APS diagnostic delay in the last years, this aspect still remains a main issue to be addressed. The time between the clinical manifestations’ onset and the diagnosis, and the risk of a misdiagnosis have to be further reduced, in order to avoid a delayed management of the patients that can be particularly damaging for their health. Indeed in our experience, even considering the limited sample size of our enquired cohort, patients had to consult multiple physicians to come to a defined diagnosis. With this in mind, national health systems might require the establishment and promotion of national and supranational networks for the study, identification, classification and sharing of knowledge on rare diseases, especially among clinicians (both general practitioners and specialized physicians) [14].

Lastly, questionnaire respondents showed a clear willing of building a critical consciousness on their disease, stressing the necessity of being educated and understood by their physicians. The importance of a rich interplay between different physicians and, at the same time, between clinicians and patients had been addressed in other chronic conditions as systemic lupus erythematosus [15], proving its efficacy in improving patients compliance, quality of life and pain control, strengthening patient-physician relationship.

Some limitations should be acknowledged. Data collection in register-based studies suffers from many limitations: necessary information may be unavailable, data collection is not performed by the researcher, confounder information is lacking, information on data quality might be missing andpotential risk of data dredging. All the above applied to our study. Similarly, no comparative data are available. Also, no analysis was made regarding the respondent’s place of residence (village/city). The authors did not analyse the distance the patient had between the hospital and the place of residence. The before mentioned methodological limitations, however, concern most studies (both single- and multi-centre ones).

While the diagnostic delay of APS has been reduced during the last years, it is still an important issue that remains to be addressed when it comes to low prevalence diseases. Joint international efforts are needed in order to improve the current knowledge on APS and ultimately to improve patients’ management and quality of life.

## Abbreviations

APS: Antiphospholipid Syndrome
PM: Pregnancy morbidity
aPL: Antiphospholipid Antibodies
S.D.: Standard Deviation
